# Experimental Evidence for the Effects of Calcium and Vitamin D on Bone: A Review

**DOI:** 10.3390/nu2091026

**Published:** 2010-09-17

**Authors:** Howard A. Morris, Peter D. O’Loughlin, Paul H. Anderson

**Affiliations:** 1 School of Pharmacy and Medical Sciences, University of South Australia, Adelaide, South Australia 5001, Australia; 2 Endocrine Bone Research Laboratory, Hanson Institute, SA Pathology, Adelaide, South Australia 5000, Australia; Email: peter.oloughlin@health.sa.gov.au (P.D.O.L.); 3 Chemical Pathology, SA Pathology, Adelaide, South Australia 5000, Australia; Email: paul.anderson@health.sa.gov.au (P.H.A.)

**Keywords:** osteomalacia, osteoporosis, dietary calcium, vitamin D, oophorectomy, bone architecture, bone strength, calcium balance

## Abstract

Animal models fed low calcium diets demonstrate a negative calcium balance and gross bone loss while the combination of calcium deficiency and oophorectomy enhances overall bone loss. Following oophorectomy the dietary calcium intake required to remain in balance increases some 5 fold, estimated to be approximately 1.3% dietary calcium. In the context of vitamin D and dietary calcium depletion, osteomalacia occurs only when low dietary calcium levels are combined with low vitamin D levels and osteoporosis occurs with either a low level of dietary calcium with adequate vitamin D status or when vitamin D status is low in the presence of adequate dietary calcium intake. Maximum bone architecture and strength is only achieved when an adequate vitamin D status is combined with sufficient dietary calcium to achieve a positive calcium balance. This anabolic effect occurs without a change to intestinal calcium absorption, suggesting dietary calcium and vitamin D have activities in addition to promoting a positive calcium balance. Each of the major bone cell types, osteoblasts, osteoclasts and osteocytes are capable of metabolizing 25 hydroxyvitamin D (25D) to 1,25 dihydroxyvitamin D (1,25D) to elicit biological activities including reduction of bone resorption by osteoclasts and to enhance maturation and mineralization by osteoblasts and osteocytes. Each of these activities is consistent with the actions of adequate circulating levels of 25D observed *in vivo*.

## 1. Introduction

Experimentation on a variety of animal models for over 80 years has clearly demonstrated that low dietary calcium, particularly in adult animals, produces osteoporosis, defined as a low quantity of normally mineralized bone. In contrast, vitamin D deficiency, especially in young animals, produces osteomalacia, defined as a defect in bone mineralization. Both osteoporosis and osteomalacia compromise the strength of bone and in humans increase the risk of fracture. They can occur in concert although they arise by distinct etiologies, a fact which has not always been recognized, particularly with regard to interpretation of the effects of vitamin D deficiency on bone structure. Severely vitamin D deficient animals are hypocalcemic, hypophosphatemic and demonstrate secondary hyperparathyroidism resulting in osteomalacia. Dietary studies with genetically modified mouse models in which vitamin D activity has been ablated indicate that osteomalacia can be resolved with a diet high in calcium and phosphate, which normalizes the plasma calcium and phosphate levels. Thus currently the major question with regard to vitamin D activity and bone tissue is whether vitamin D can act directly on bone cells to modulate skeletal health.

## 2. Dietary Calcium Deficiency

Studies from the early decades of the 20th Century identified that various animal models fed a diet low in calcium demonstrated a negative calcium balance and gross bone loss [1]. Bauer and colleagues studied the effects of low calcium diets on cats by visual inspection of longitudinal sections of the humerus [1]. They found that significant bone loss was evident from the trabecular bone compartment in contrast to cortical bone and concluded that the trabecular compartment of bone tissue was the metabolically most active compartment of the skeleton. Using the technique of bone ash weight Hodgkinson and colleagues could only detect significant bone loss when calcium deficiency was combined with estrogen deficiency following oophorectomy [2]. However with the availability of 2-dimensional bone histomorphometry, bone losses, quantified as a reduction of trabecular bone volume as a fraction of total volume (BV/TV) in the trabecular compartment and a reduction of cortical bone volume in the cortical compartment, were detected following dietary calcium deprivation while with oophorectomy bone loss was only detectable in the trabecular bone compartment [3]. Their studies identified that bone loss with estrogen deficiency was the result of perforation and dissolution of individual trabeculae and the conversion of trabecular plates into rods as a result of a marked increase in osteoclastic bone resorption. They also confirmed that the combination of calcium deficiency and oophorectomy enhanced overall bone loss. 

Shen and colleagues noted difficulties with conducting studies on rat models including the uncertainty of the calcium requirement for mature rats and therefore difficulty estimating what constituted high and low dietary contents. As well the impact of growth possibly confounded the use of rodents in such studies. Careful calcium balance data were later published indicating that mature ovary-intact, 6 month-old Sprague-Dawley female rats required 0.2% dietary calcium to remain in balance, that is a 0.2% calcium intake was sufficient to balance for losses of calcium in the urine and faeces [4]. Following oophorectomy the dietary calcium intake to remain in balance increases to over 1% with an estimate of approximately 1.3% dietary calcium required to remain in balance [4]. These important data clearly identified that diets below 0.2% placed ovary-intact, adult female rats in a negative calcium balance, with losses of calcium in the urine and faeces being greater than the dietary intake of calcium and therefore under these conditions it is necessary to mobilise calcium from the skeleton to maintain plasma calcium homeostasis. Baldock and colleagues carefully characterized the femoral growth characteristics of female Sprague-Dawley rats reporting that growth was markedly slowed between 5 and 7 months of age and was undetectable after 12 months of age despite the presence of a growth plate until at least 18 months of age [5]. These data have improved the adult rat model for the study of hormonal and dietary factors on bone mineral homeostasis. However dietary calcium requirements for rats during growth, pregnancy or lactation remain to be carefully quantified and are likely to be greater than for adult animals.

## 3. The Interaction between Estrogen and Dietary Calcium Deficiencies

In the adult rat, estrogen deficiency as a result of oophorectomy, initially stimulates osteoclastogenesis and bone resorption, which is detectable at 6 days post operation. This is followed by an increase in bone formation markers at 9 days with trabecular bone loss not being detectable before 15 days post oophorectomy [6]. This increase in bone cell activity is likely to be the sum of the effects resulting from the removal of the suppressive effect that estrogen exerts on bone cells to reduce expression of RANKL and other cytokines which stimulate osteoclastogenesis [7] and the requirements to maintain blood calcium homeostasis in the face of a negative calcium balance. The contribution of each of these factors is yet to be quantified.

**Figure 1:**
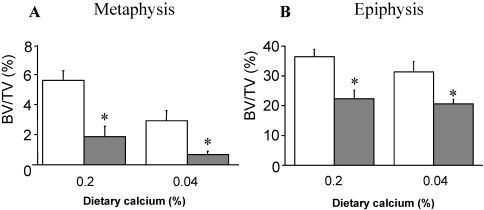
Trabecular bone volume (BV/TV) in the distal femora from 10-month old Sprague-Dawley rats following 4 months of dietary calcium feeding and 3 months post-operation: (**A**) Metaphyseal region; (**B**) Epiphyseal region. White bars: ovary-intact, sham operated; Grey bars: oophorectomized.

Estrogen deficiency in the adult rat model fed a 0.8–1% calcium diet stimulates trabecular bone loss in the distal femoral metaphysis and diaphysis regions but not in the epiphysis [8,9]. However when dietary calcium is reduced to 0.2% bone loss following oophorectomy is detectable in each of these three regions [10]. Extra bone loss occurs in the metaphysis when diet calcium is further reduced to 0.04% in ovary-intact rats but no further loss occurs in the epiphysis (Figure 1). 

**Figure 2:**
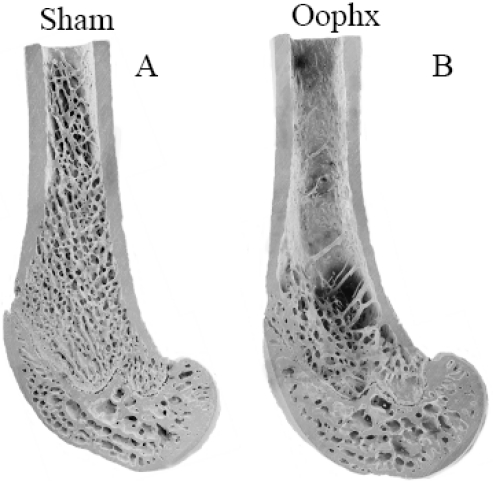
Scanning electron micrographs of distal femora from 11-month old Sprague-Dawley female rats fed 0.8% diet calcium at 150 days post-operation: (**A**) Ovary-intact, sham operation; (**B**) Oophorectomized. In the Diaphyseal and Metaphyseal region bone loss is evident following oophorectomy which is absent in the epiphysis.

Thus despite highly similar architecture amongst these three trabecular bone regions there is a differentiation with regard to response to increased osteoclastogenesis and rate of bone loss when dietary calcium is limiting [8]. Trabecular bone in the epiphysial region, which is encased in a thin cortex, experiences considerably higher mechanical forces than other regions (Figure 2). In contrast trabeculae in the metaphysis and particularly the diaphysis are encased in thicker cortices and therefore experience significantly reduced mechanical forces. These data strongly suggest that during estrogen depletion regions of bone which experience greater mechanical forces are preferentially maintained over the regions of bone which experience a reduced mechanical load. This occurs despite the increased bone cell activities due to the negative calcium balance. However, over an extended duration of estrogen depletion or further reduction in dietary calcium intake, bone resorption in these regions of high mechanical load occur, presumably following the resorption of bone from the readily accessible stores (Lee MC, O’Loughlin PD, Anderson PH, unpublished data).

The importance to bone health of maintaining a positive calcium balance in the presence of high bone turnover such as with estrogen deficiency is demonstrated with very interesting preliminary data we have recently obtained. Adult female Sprague-Dawley rats, fed 0.4% calcium, were oophorectomized at 3 months of age, losing bone over a further 3 months. These animals were then placed on diets varying in calcium from 0.4% to 1.6%, the latter diet providing sufficient calcium for estrogen-deficient rats to attain a positive calcium balance. At 9 months of age, after a further 3 months on these diets trabecular bone volume had returned to the levels of ovary-intact animals maintained on diets providing a positive calcium balance [11]. Most interesting from this study was the finding that the oophorectomized animals fed the highest diet calcium demonstrated the lowest activation frequency for bone turnover, the highest mean wall thickness and the longest formation period. These data suggest that a dietary calcium level sufficient to attain a positive calcium balance can modulate bone cell activity to reduce bone turnover with an extension of the time during whichosteoblasts form new bone within the basic multicellular unit during each bone turnover event.

## 4. The Interaction between Vitamin D and Dietary Calcium Deficiencies

It has long been established that marked vitamin D deficiency results in osteomalacia, a bone mineralization defect associated with hypocalcemia, hypophosphataemia and hyperparathyroidism [12]. However the relationship between low vitamin D status and osteoporosis, a condition with normal mineralization but low bone volume, has also been claimed to result from depleted vitamin D levels. A report from the 1970’s that patients with hip fractures have a depleted vitamin D status [13] was confirmed at a temperate latitude [14] where a mean serum 25D value of 40 nmol/L for hip fracture patients was found. There is strong evidence of the association between a low vitamin D status and low bone mineral density [15] however the nature of the bone defect has not been clearly defined as hip fractures and low bone density can result from either osteoporosis or osteomalacia. 

**Figure 3:**
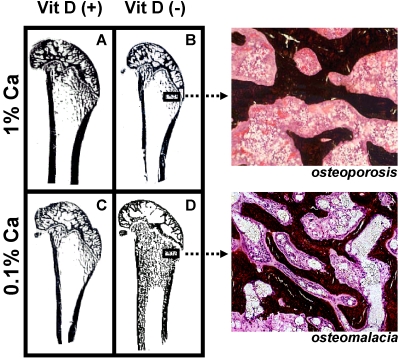
Longitudinal sections (Von Kossa stained) of 9-month old Sprague-Dawley rat distal femora following 3 months feeding either (**A**) 1% calcium/20 IU vitamin D_3_/day; (**B**) 1% calcium/0 IU vitamin D_3_/day; (**C**) 0.1% calcium/20 IU vitamin D_3_/day; (**D**) 0.1% calcium /0 IU vitamin D_3_/day. Highly trabecularized bone with osteomalacia (D) is evident in contrast to reduced trabecular bone volume (B &C) compared to (A).

Variation of dietary vitamin D and calcium levels fed to adult female rats has provided very interesting data as to the differential effects of deficient levels of these nutrients. Rats were allocated to either vitamin D deficient or replete diets and then further allocated to 0.1% or 1% calcium diets for 3 months. Rats fed the vitamin D deplete diet achieved mean serum 25-hydroxyvitamin D (25D) levels of 14 nmol/L while animals fed vitamin D replete diets achieved 25D levels of 97 to 135 nmol/L [16]. Osteoid maturation time (OMT) was significantly higher (12.6 days) in the vitamin D deplete/0.1% calcium group, a result which is diagnostic for osteomalacia. In the remaining groups OMT was approximately 6 days therefore excluding osteomalacia. However metaphyseal trabecular bone volume and cortical width were reduced in both vitamin D replete/0.1% calcium and vitamin D deplete/1% calcium groups compared with rats fed the vitamin D replete/1% calcium (Anderson PH, Iida S, Morris HA, unpublished data, Figure 3). These data indicate that in the context of vitamin D and dietary calcium depletion, osteomalacia occurs only when both vitamin D and dietary calcium levels are markedly reduced and osteoporosis occurs with either a low calcium diet alone or when vitamin D status is low and dietary calcium is adequate. 

A major controversy in this field is the suggestion that vitamin D receptor or vitamin D activity within bone cells is redundant for normal bone health. The controversy has arisen because osteomalacia, whether as a result of nutritional deficiency of vitamin D or genetic ablation of vitamin D activity can be resolved by nutritional supplementation of calcium and phosphate alone [17,18]. Statements have been made that such “rescue” diets return the bone structure to “normal” [18]. However these studies particularly with genetically modified mice have often been completed at an age of less than 12 weeks when growth continues at a relatively high rate. One study with genetically modified mice, in which either the vitamin D receptor (*Vdr*) gene or the 25-hydroxyvitamin D-1α-hydroxylase (*Cyp27b1*) genes were ablated, was continued until mice were 16 weeks of age [19]. They found that while the genetically modified mice fed the rescue diet had resolved the osteomalacia, the femoral metaphyseal trabecular bone volume was only approximately 50% of that in the age- and sex-matched wild type mice. These data suggest that in addition to its action to stimulate intestinal calcium absorption vitamin D activity is also required for the normal function of cells involved in bone turnover. New models of cell-specific ablation of the *Vdr* are required to elucidate the relative roles of vitamin D activity within the bone micro-environment.

As discussed above increased risk of hip fracture in the elderly is associated with mean serum 25D values of approximately 40 nmol/L [12]. Furthermore clinical studies indicate that the risk of non-vertebral fractures including the hip does not reduce until serum 25D levels of 80 nmol/L or greater are achieved [20]. However the effects on bone cell activities of such levels of vitamin D are unknown. Rodent studies have been most useful for the investigation of this issue. Young adult rats (3 months of age) fed a 0.4% calcium, vitamin D deficient diet achieved a mean serum 25D level of 12 nmol/L with an OMT indicative of osteomalacia [21]. When serum 25D was raised to 22 nmol/L osteomalacia was resolved while osteoporosis was evident in the groups with serum 25D levels between 22 and 80 nmo/L. This loss of trabecular bone volume was due to increased bone resorption as a result of increased expression of the *RankL* gene in bone and increased osteoclastogenesis. No relationship was found between bone volume and either serum 1,25D or parathyroid hormone [21]. Further studies have been conducted with 15 month old rats comparing the effects of varying vitamin D status on 0.1% or 1% calcium diets (Lee MC, Anderson PH, O’Loughlin PD unpublished results). The positive relationship between trabecular bone volume and serum 25D levels was confirmed and extended to a similar relationship between cortical bone volume and 25D levels with a maximum cortical bone volume being achieved at 25D levels of 100 nmol/L or greater. Furthermore our studies indicate that increasing 25D levels to greater than 80 nmol/L was effective in increasing cortical bone volume only in animals achieving a positive calcium balance. Consistent with the increased cortical bone volume, increased bone strength was only achieved in animals fed 1% calcium and serum 25D levels greater than 80 nmo/L. Therefore for rodent models osteomalacia occurs when fed calcium diets at or below the level required to achieve a positive calcium balance and serum 25D levels are below 20 nmol/L. When 25D levels are between 20 and 80 nmol/L bone loss occurs at both the trabecular and cortical compartments. Maximum bone architecture and strength is only achieved when an adequate vitamin D status is combined with sufficient dietary calcium to achieve a positive calcium balance.

As discussed above these data indicate that the major circulating vitamin D metabolite associated with increased bone structure and strength is the pro-hormone 25D and not the active metabolite 1,25D. To investigate whether normal levels of serum 1,25D can act in the same manner as serum 25D to suppress osteoclastogenesis and bone resorption, adult female rats with 25D levels at approximately 20 nmol/L were infused with 1,25D. In contrast to the suppression of the expression of the *RankL* gene in bone by increasing circulating 25D, 1,25D infusion increased the expression of the *RankL* gene in bone with no detectable suppression of osteoclasts over the 14 days of the infusion [Sawyer R, Anderson PH, Morris HA unpublished results]. Thus experimental data strongly indicate that the circulating levels of the pro-hormone 25D are necessary to optimise bone architecture rather than the active metabolite 1,25D. The question as to what is the biological activity of 25D in bone cells is of considerable interest.

## 5. Endogenous Metabolism of Vitamin D within Bone Cells

Each of the major bone cells, osteoblasts, osteoclasts and osteocytes are capable of metabolizing 25D to 1,25D to elicit biological activities. Human and rodent osteoblasts strongly express the 25-hydroxyvitamin D-1,α-hyroxylase (CYP27B1) enzyme which is essential to convert 25D to 1,25D and to increase expression of key genes associated with maturation and mineralization [22]. 25D in the osteoblast culture media reduces cell proliferation and stimulates osteoblast maturation and mineralization *in vitro.* More recently, it has been demonstrated that *Cyp27b1* expression in pre-osteoclast cells is also necessary for 25D to increase osteoclastogenesis [23]. Interestingly osteoclasts formed in the presence of 25D demonstrated reduced resorbing activity than cells matured in the absence of vitamin D metabolites or cells in which the *Vdr* gene has been ablated. Osteocytes also express the C*yp27b1* gene and mRNA levels increase with differentiation and are associated with the acquisition of mature osteocyte genes including *Mepe (Matrix extracellular PhosphoglycoprotEin)*, *Dmp1 (Dentin matrix protein 1)*, *Phex (Phosphate-regulating gene with Homologies to Endopeptidase on the X chromosome)* (Atkins GJ, unpublished data) and *Fgf23 (fibroblast growth factor 23)* [24]. Thus *in vitro* data provide strong evidence that the pro-hormone 25D is capable of metabolism by bone cells to the active hormone 1,25D to elicit various activities including the reduction of bone resorption by osteoclasts and to enhance maturation and mineralization by osteoblasts and osteocytes. Each of these activities is consistent with the actions of adequate circulating levels of 25D observed *in vivo*.

As discussed above the anabolic effects of circulating 25D on the skeleton are dependent on a dietary calcium intake sufficient to ensure a positive calcium balance that is a dietary calcium intake which provides a greater amount of calcium than is excreted from the body via the urine and faeces. The question arising from such findings is whether calcium exerts any biological activities within the skeletal system rather than simply providing a raw material, in conjunction with phosphate, for mineralization. *In vitro* studies indicate that the addition of calcium to culture media enhances the ability of osteoblast-like cells to mineralize *in vitro* (Yang D, Atkins GJ, Morris HA unpublished results). Interestingly a recent report describes that when rats are fed a dietary calcium level sufficient to ensure a positive calcium balance, the levels of *Cyp27b1* mRNA are 3-fold higher in bone tissue compared with animals fed a low calcium diet [16]. Messenger RNA levels for *Cyp24*, which codes for the vitamin D catabolic enzyme 25-hydroxyvitamin D 24-hydroxylase and is highly regulated by 1,25D were also elevated in bone from these animals suggesting that 1,25D levels are also higher in the bone tissue. Thus, a contributing effect of adequate dietary calcium intake to their positive effects on bone mineral homeostasis may be, at least in part, through increasing the synthesis of 1,25D within bone tissue.

## 6. Conclusions

Studies in animals have identified that feeding low levels of dietary calcium produces a negative calcium balance and gross bone loss. The calcium requirement in the estrogen deficient state is markedly increased and therefore the combination of calcium deficiency and oophorectomy enhances overall bone loss. In the context of vitamin D and dietary calcium depletion, osteomalacia occurs only when both vitamin D and dietary calcium levels are reduced and osteoporosis occurs with either a low calcium diet in the presence of adequate vitamin D or when vitamin D status is low in the presence of adequate dietary calcium. Maximum bone architecture and strength is only achieved when an adequate vitamin D status is combined with sufficient dietary calcium to achieve a positive calcium balance. Each of the major bone cells, osteoblasts, osteoclasts and osteocytes are capable of metabolizing 25D to 1,25D to elicit biological activities including reduction of bone resorption by osteoclasts and to enhance maturation and mineralization by osteoblasts and osteocytes. Thus optimal bone health is only achieved when the vitamin D status is improved thus providing adequate levels of the circulating pro-hormone 25D for local metabolism by bone cells into the active hormone 1,25D. Each of these bone cell activities is consistent with the actions of adequate circulating levels of 25D observed *in vivo* and with clinical outcomes from vitamin D and calcium dietary supplementation.

Valuable data have been derived from animal experimentation on the physiological responses of bone mineral homeostasis to variations in dietary calcium intake and vitamin D status in both the context of estrogen sufficiency and deficiency. Such data provide support for clinical studies, often derived from epidemiological or case control study formats, indicating that nutritional deficiencies of calcium and vitamin D, especially in postmenopausal women, produce adverse bone health outcomes and increase the risk of osteoporotic fractures. Animal studies provide research models for investigating the mechanisms by which such adverse outcomes may arise allowing the combined investigation of bone architecture, bone cell activities and molecular changes in response to nutritional manipulation. Knowledge of the molecular and cellular basis is essential for defining the nutritional levels required for optimal health outcomes, for acceptance by the clinical community of the essential requirements for calcium and vitamin D nutrition and for the development of public health policies aimed at osteoporosis prevention. 
